# Multi-Echo-Based Echo-Planar Spectroscopic Imaging Using a 3T MRI Scanner

**DOI:** 10.3390/ma4101818

**Published:** 2011-10-17

**Authors:** Jon K. Furuyama, Brian L. Burns, Neil E. Wilson, M. Albert Thomas

**Affiliations:** 1 Department of Radiological Sciences, University of California, Los Angeles, CA 90095, USA; 2 Medical Imaging Informatics (MII) Lab, University of California, Los Angeles, CA 90095, USA

**Keywords:** multi-echo, echo-planar spectroscopic imaging

## Abstract

The use of spin-echoes has been employed in an Echo-Planar Spectroscopic Imaging (EPSI) sequence to collect multiple phase encoded lines within a single TR in a Multi-Echo-based Echo-Planar Spectroscopic Imaging technique (MEEPSI). Despite the $T_{2}$ dependence on the amplitude of the spin-echoes, the Full Width at Half Maximum (FWHM) of the derived multi-echo Point Spread Function (PSF) is shown to decrease, indicating an improved overall spatial resolution without requiring any additional scan time. The improved spatial resolution is demonstrated in the one-dimensional (1D) spatial profiles of the N-Acetyl Aspartate (NAA) singlet along the phase encode dimension in a gray matter phantom. Although the improved spatial resolution comes at the expense of spectral resolution, it is shown *in vivo* that peak broadening due to $T_{2}^{*}$ decay is more significant than the loss of resolution from using spin-echoes and therefore does not affect the ability to quantify metabolites using the LCModel fitting algorithm.

## 1. Introduction

Chemical Shift Imaging (CSI) [1], also known as Magnetic Resonance Spectroscopic Imaging (MRSI) [2], holds great potential to noninvasively map biochemical information by means of Nuclear Magnetic Resonance (NMR). The conventional CSI sequence operates by acquiring a two-dimensional (2D) image as a series of individually phase encoded free induction decays (FIDs) or partial spin-echoes of a localized volume. The time required to sample a spatially-encoded 3D volume is thus $N_{x}\times N_{y}\times N_{z}\times \mathrm{TR}\times n$ where $N_{i}$ is the number of complex points in the *i*th spatial dimension, TR is the repetition time, and *n* is the number of averages. A coarse 2D image with a pixel resolution of 32×32×1, TR=1 s, and 1 average requires a scan time of just over 17 min. Depending on the desired voxel size, scans may require multiple averages to achieve adequate signal-to-noise ratio (SNR) which pushes scan times to undesirable levels. Without any acceleration techniques, CSI sequences are strictly limited to very low resolutions in order to maintain clinically feasible scan times.

Mansfield proposed the use of an echo-planar readout gradient train to simultaneously acquire one spatial dimension as well as one spectral dimension [3,4]. However, at that time, clinical scanners were not equipped to adequately handle the required gradient waveforms [5], and it was not for another decade until Posse *et al.* implemented the first clinically applicable Proton Echo-Planar Spectroscopic Imaging (PEPSI) [6,7] protocol, also known as Echo-Planar Spectroscopic Imaging (EPSI) by others [8]. EPSI employs a time varying readout gradient by which the same line in *k*-space is repeatedly frequency encoded, effectively encoding spatial information as a function of time and removing the need to phase encode that spatial dimension. Performing a fast Fourier transform (FFT) along the readout direction yields the spatial information, and a subsequent FFT over the repeated readouts yields spatially-encoded spectral information. The second spatial dimension is still incrementally phase encoded similar to spin-echo MRI. Because the spatial information from the readout dimension is not individually phase encoded, the same hypothetical spatio-temporal data set previously described can be collected in just 32 s, representing a reduction in scan time of well over an order of magnitude. Such a reduction of scan time makes it feasible to increase spatial resolution, collect 3D data sets [8], or even acquire spatially resolved multi-dimensional spectral data in a clinical setting [9].

Since the phase encoding direction is sampled incrementally, it is easily understood that increasing the desired spatial resolution comes at the expense of increased scan time. In other words, in order to double the resolution of a regularly sampled image, the extent of the phase-encoded *k*-space data must be doubly sampled. Spin-echoes have previously been used to accelerate CSI techniques [10,11] but to our knowledge has not been combined with a standard EPSI readout. In this work, we propose a Multi-Echo-based Echo-Planar Spectroscopic Imaging (MEEPSI) sequence which makes use of $180^{\circ }$ refocussing pulses to create spin-echoes that can be phase encoded for the collection of multiple *k*-space lines per TR with the goal of collecting more lines without increasing scan time. The use of spin-echoes results in different *k*-space lines being collected at different TEs. Therefore, the *k*-space sampling window is both $T_{2}$-weighted and *J*-modulated, which results in a modified Point Spread Function (PSF) that is shown to be a linear combination of the PSFs for the regularly sampled and doubly-sampled *k*-space windows. This introduces spectral and spatial artifacts originating from a modulation of the PSF, as both $T_{2}$ decay and evolution due to *J*-coupling are not refocussed by a $180^{\circ }$ pulse [12].

In order to minimize artifacts due to $T_{2}$ losses, the time between the different echoes needs to be kept as short as possible, which limits the number of acquired spectral points and overall spectral resolution. We show, however, that with typical *in vivo* shimming conditions and line-widths, the loss of resolution due to peak broadening exceeds that due to the reduced number of acquired spectral points. Phantom scans are presented from a gray matter phantom to study the spatial resolution enhancement, and an *in vivo* scan is presented from a healthy human volunteer to show that quantitation using the LCModel fitting algorithm [13] is not deteriorated due to the loss of spectral resolution.

## 2. Theory

### 2.1. Multi-Echo Sequence

**Figure 1:**
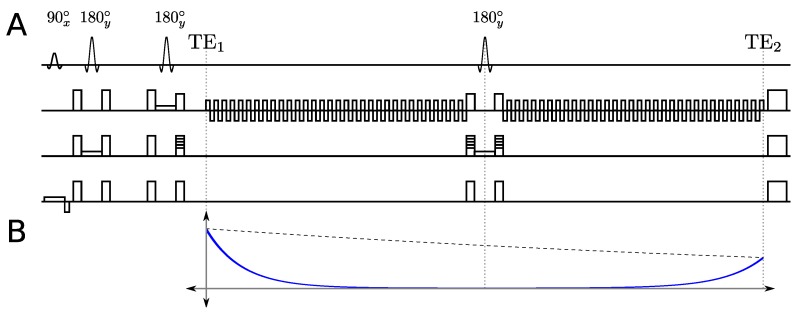
(**A**) An illustration of the pulse sequence of the PRESS-based MEEPSI sequence, showing the three localization pulses and two echo-planar readouts separated by a slice selective $180^{\circ }$ pulse; (**B**) Diagram showing the effect of both $T_{2}$ (dashed line) and $T_{2}^{*}$ (solid line) decay on the overall shape of the signal envelope.

The MEEPSI technique employs two individually phase encoded EPSI readouts separated by a slice selective refocusing $180^{\circ }$ pulse. For a review of the basic principles behind the EPSI sequence, please refer to the review by Mulkern [14]. An illustration of the MEEPSI sequence can be seen in Figure 1(A). A typical MEEPSI sequence involves either a Point RESolved Spectroscopy (PRESS) [15] or a STimulated Echo Acquisition Mode (STEAM) [16] excitation scheme to localize the volume of interest (VOI). The VOI can also be excited using a slice-based excitation scheme as implemented by others [8]. Following the last localization pulse, the magnetization is phase encoded and recorded by the first EPSI readout. The standard EPSI readout uses a repeating pair of readout gradients of opposite polarity to repeatedly frequency encode the same line in *k*-space. This repetition records the spatial information as a function of time, effectively interleaving the simultaneous collection of one spatial and one spectral dimension. Once the first EPSI readout is complete, the initial phase encoding is reversed, and the magnetization is refocused with a slice-selective $180^{\circ }$ pulse. The magnetization is then phase encoded to another line in *k*-space and subsequently measured with the second EPSI readout.

The bipolar nature of the EPSI readout gradients results in two different *k*-space trajectories, the first from $-k_{max}\to k_{max}$, and the second from $k_{max}\to -k_{max}$, which produce two mirror images when the FFT is taken along the readout dimension. This situation is remedied by time-reversing the second echo in each readout pair of the first echo-planar readout, yielding the same image as from the first gradient echo. Since the echo-planar readout is repeated multiple times, all even-numbered echoes from the first readout need to be time reversed to match the odd-numbered echoes. Once corrected, both even and odd echoes can be added together to increase the SNR by a factor of $\sqrt{2}$, which comes at the expense of half of the spectral bandwidth. The action of a $180^{\circ }$ pulse is to reverse any evolution stemming from the linear terms in the Hamiltonian (chemical shift, field inhomogeneity, *etc*.) and can be thought of as a pseudo time-reversal operator. As a result, the first gradient echo after the $180^{\circ }$ pulse corresponds to the same trajectory in *k*-space as the final [even-numbered] gradient echo in the first echo-planar readout. This requires the polarity of the first echo in the second echo-planar readout to be reversed, and consequently, all the odd-numbered echoes after the $180^{\circ }$ pulse need to be time reversed.

**Figure 2:**
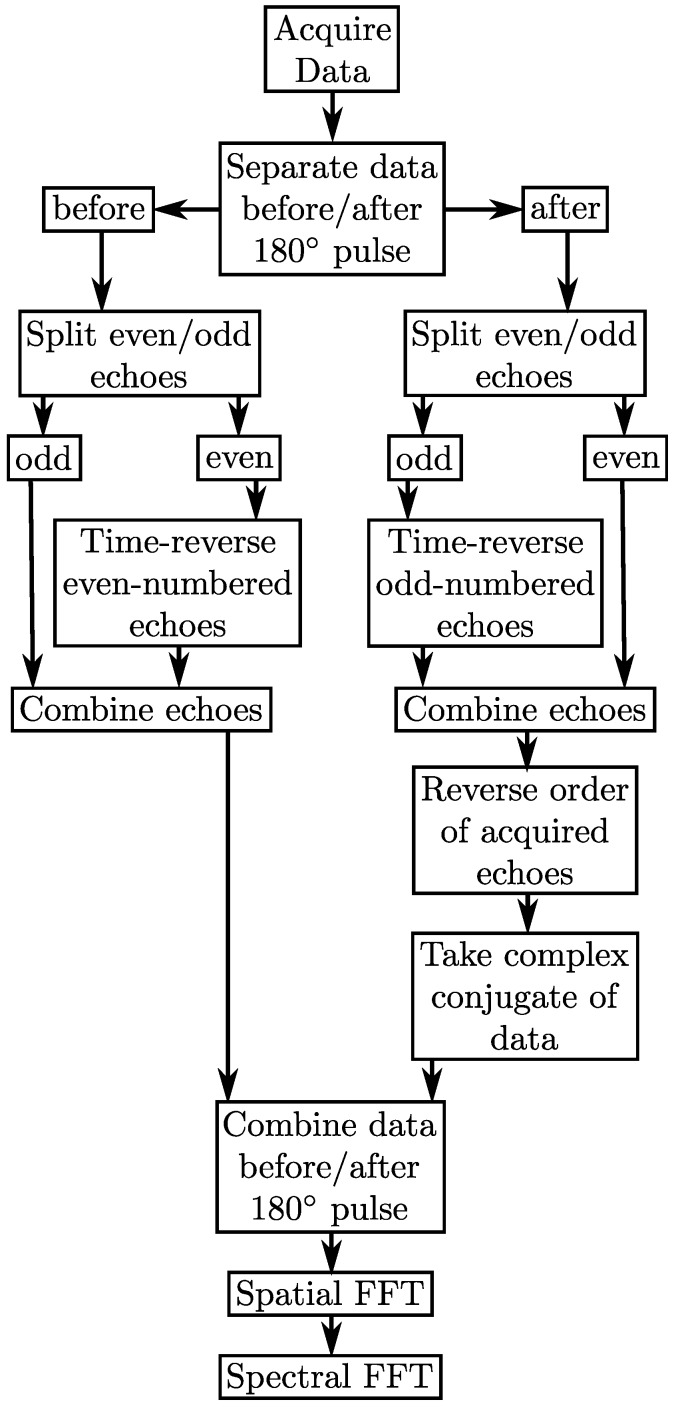
Flowchart showing how to separate and process the different echoes in the MEEPSI sequence as shown in Figure 1.

As shown in Figure 1(B), by placing the second EPSI readout after the $180^{\circ }$ pulse, the evolution that is recorded is from the rephasing of the magnetization (first half of ${\mathrm{TE}}_{2}$), which is in contrast to the first EPSI readout where the signal is dephasing (second half of ${\mathrm{TE}}_{1}$). Since the peak of the magnetization of the second EPSI readout occurs at the end, as opposed to the beginning in the first EPSI readout, the ordering of the second EPSI readout needs to be reversed so that all echo maximums line up together in *k*-space. This reordering of the second EPSI readout serves as a time-reversal which has the effect of mirroring the spectral content. Therefore, the complex conjugate of the second EPSI readout must be taken after the order has been reversed. These steps are summarized in the flowchart in Figure 2.

### 2.2. Multi-Echo PSFs

**Figure 3:**
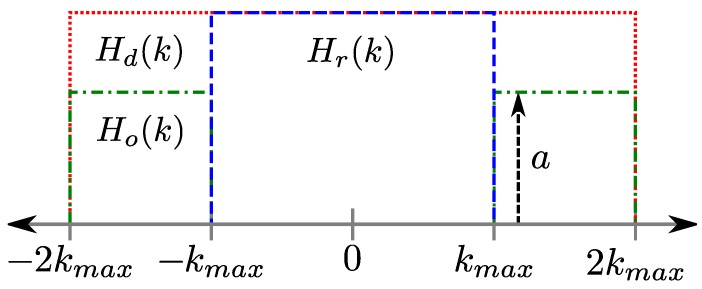
Different weighting functions for the sampling of *k*-space, where $H_{r}\left(k\right)$ represents the regularly-sampled *k*-space window from a single-echo sequence, $H_{d}\left(k\right)$ represents the doubly-sampled *k*-space window for twice the resolution, and $H_{o}\left(k\right)$ represents the outer half of *k*-space that would be sampled by the second echo in a multi-echo sequence with an amplitude of $a\;=\;\exp\{-\left(1/T_{2}+i2\pi J\right)\Delta {\mathrm{TE}}_{21}\}$.

The use of multiple spin-echoes within a single TR is equivalent to filling *k*-space with different TEs. In the absence of $T_{2}$ relaxation and *J*-coupling, the MEEPSI sequence (or other multi spin-echo sequences) can improve the spatial resolution of standard CSI sequences without any artifacts. However, both $T_{2}$ relaxation and *J*-evolution originate from terms in the Hamiltonian that are not refocused by a $180^{\circ }$ pulse, resulting in an amplitude and phase modulation in the *k*-space encoding that can lead directly to both spectral and spatial Gibbs ringing in the data [12]. Therefore, the complex magnetization, $M_{xy}=M_{x}+iM_{y}$, at ${\mathrm{TE}}_{2}$ with respect to the magnetization at ${\mathrm{TE}}_{1}$ can be informally described as

(1) $${\vec{M}}_{xy}\left({\mathrm{TE}}_{2}\right)={\vec{M}}_{xy}\left({\mathrm{TE}}_{1}\right)\times \exp\left\{-\left(\frac{1}{T_{2}}+i2\pi J\right)\Delta {\mathrm{TE}}_{21}\right\}$$

where $\Delta {\mathrm{TE}}_{21}={\mathrm{TE}}_{2}-{\mathrm{TE}}_{1}$. For the variety of different *J*-coupled metabolites, both strongly and weakly coupled, the signal behavior for each specific metabolite can become quite complicated and so the use of Equation (1) is meant simply to illustrate both the phase and amplitude modulated behavior of using spin-echoes. This modulation affects the *k*-space sampling window for $M_{xy}\left({\mathrm{TE}}_{2}\right)$, so care must be taken in determining which *k*-space lines are encoded by the first and second EPSI readouts. For the remainder of this discussion, the phase encoded EPSI readouts after the $180^{\circ }$ pulse are placed on the edges of *k*-space as shown in Figure 3.

In a single-echo experiment, the regularly sampled *k*-space window, $H_{r}\left(k\right)$, can be described as

(2) $$H_{r}\left(k\right)=\Pi \left(\frac{k}{2k_{max}}\right)$$

where

(3) $$\Pi \left(k\right)=\left\{\begin{array}{cc} 1 & \mathrm{for}|k|<1/2\\ 0 & \mathrm{otherwise} \end{array}\right.$$

is the rect function with unit width, and its Fourier transform

(4) F{Π(k)}=sinc(πx)

yields the familiar sinc-shaped PSF

hr(x)=FHr(k)(5)=2kmaxsinc(2πkmaxx)

In order to double the spatial resolution, the required *k*-space coverage needs to be doubled as in Figure 3, which produces the doubly-sampled *k*-space window, $H_{d}\left(k\right)$, defined as

(6) $$H_{d}\left(k\right)=\Pi \left(\frac{k}{4k_{max}}\right)$$

with its corresponding PSF

hd(x)=F{Hd(k)}(7)=4kmaxsinc(4πkmaxx)

where it can be seen that the PSF in Equation (7) has twice the amplitude and half the width of the PSF described by Equation (5), indicating twice the resolution. The resolution of the PSF in Equation (5) can be enhanced by filling the region between $k_{max}$ and $2k_{max}$ with signal acquired from a spin echo. As shown in Figure 3, the resulting multi-echo *k*-space coverage, $H_{m}\left(k\right)$ can be defined as

(8) $$H_{m}\left(k\right)=\left\{\begin{array}{cc} 1 & \mathrm{for}|k|<k_{max}\\ a & \mathrm{for}k_{max}<\left|k\right|<2k_{max}\\ 0 & \mathrm{otherwise} \end{array}\right.$$

where

(9) $$a=\exp\left\{-\left(\frac{1}{T_{2}}+i2\pi J\right)\Delta {\mathrm{TE}}_{21}\right\}$$

and is the result of the modulation of $M_{xy}\left({\mathrm{TE}}_{1}\right)$. Calculating the PSF for Equation (8) can be simplified by redefining the multi-echo window such that

(10) $$H_{m}\left(k\right)=H_{r}\left(k\right)+H_{o}\left(k\right)$$

where $H_{o}\left(k\right)$ is the region of *k*-space beyond $k_{max}$ as seen in Figure 3, and can be defined as

(11) $$H_{o}\left(k\right)=a\left[H_{h}\left(k-\frac{3}{2}k_{max}\right)+H_{h}\left(k+\frac{3}{2}k_{max}\right)\right]$$

where $H_{h}\left(k\right)$ has half the width of $H_{r}\left(k\right)$ and is defined as

(12) $$H_{h}\left(k\right)=\Pi \left(\frac{k}{k_{max}}\right)$$

Using the modulation property of the Fourier transform

(13) $$s\left(x\right)\cos\left(2\pi k_{0}x\right)\overset{F}{⟷}\frac{1}{2}\left[S\left(k-k_{0}\right)+S\left(k+k_{0}\right)\right]$$

the PSF for $H_{o}\left(k\right)$ is

ho(x)=F{Ho(k)}(14)=2akmaxsinc(πkmaxx)cos(3πkmaxx)

which can be simplified using the trigonometric identity

(15) $$\sin u\cos v=\frac{1}{2}\left[\sin(u+v)+\sin(u-v)\right]$$

which produces

ho(x)=2aπxsin(πkmaxx)cos(3πkmaxx)=aπxsin(4πkmaxx)+sin(-2πkmaxx)=akmax4sinc(4πkmaxx)-2sinc(2πkmaxx)(16)=ahd(x)-hr(x)

As a result, it can be seen that the PSF for Equation (10) is simply

hm(x)=F{Hr(k)+Ho(k)}=hr(x)+ho(x)(17)=(1-a)hr(x)+ahd(x)

which is a linear combination of the regularly-sampled and the doubly-sampled PSF.

**Figure 4:**
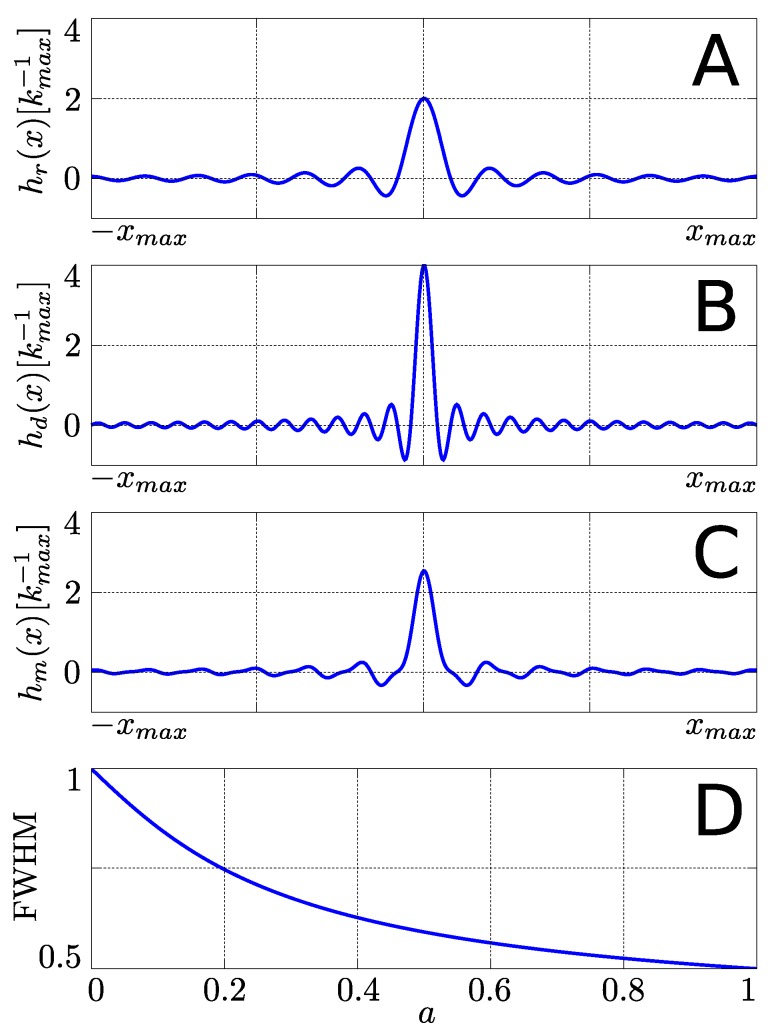
Graphical display of (**A**) the regularly sampled PSF, $h_{r}\left(x\right)$ as shown in Equation (5); (**B**) the doubly-sampled PSF, $h_{d}\left(x\right)$ as in Equation (7); and (**C**) the multi-echo PSF, $h_{m}\left(x\right)$, as shown in Equation (17) with values of $T_{2}\;=\;\Delta {\mathrm{TE}}_{21}$ and J=0 such that $a=e^{-1}$; (**D**) The dependence of the normalized (with respect to $h_{r}\left(x\right)$) FWHM for $h_{m}\left(x\right)$ as a function of real values of *a* (Equation (9))

Figure 4 shows the different PSFs. The familiar sinc-shaped PSF for the regularly sampled phase encoding sampling scheme, $h_{r}\left(x\right)$, can be seen in Figure 4(A). As expected, when twice as much *k*-space is covered, the resolution is seen to increase as the width of the PSF decreases in Figure 4(B). As shown in Equation (17), the PSF for the multi-echo sequence is a linear combination of Figure 4(A,B), which is dependent on the value for *a*. As an example, $h_{m}\left(x\right)$ is plotted in Figure 4(C) for $\Delta {\mathrm{TE}}_{21}=T_{2}$ and J=0 such that $a=e^{-1}\approx 0.368$. The height of $h_{m}\left(x\right)$ is seen to be greater than that of $h_{r}\left(x\right)$, and the FWHM is narrower as well.

The FWHM for Equation (17) cannot be algebraically determined and must be determined numerically. Figure 4(D) shows the numerically calculated values for the FWHM, normalized with respect to $h_{r}\left(x\right)$, as a function of real values of *a*. As a→0, it can be seen that $h_{m}\left(x\right)\to h_{r}\left(x\right)$, which yields a normalized FWHM of 1. At the other extreme, as a→1 it can be seen that $h_{m}\left(x\right)\to h_{d}\left(x\right)$ with a FWHM that is half that of $h_{r}\left(x\right)$, corresponding to the doubled resolution of $h_{d}\left(x\right)$. One thing to note about the dependence of the FWHM as a function of *a* is that Figure 4(D) is steeper at the beginning for smaller values of *a* than it is for larger values of *a*. This shows that for even the smallest values of *a*, there is a noticeable gain in resolution. For example, a value as low as a≈0.2 in Figure 4(D) shows the resolution has already improved by 50%. From Equation (9) it can be seen that *a* can be increased by decreasing $\Delta {\mathrm{TE}}_{21}$. However, by decreasing $\Delta {\mathrm{TE}}_{21}$, the total number of spectral points that can be sampled in each EPSI readout is reduced, which reduces the collected spectral resolution.

The discussion up to this point has been limited to only real values of *a*. As can be seen in Equation (9), for metabolites that are *J*-coupled, *a* can be complex valued, acquiring a phase of $\phi =2\pi J\Delta {\mathrm{TE}}_{21}$. This leads to a complex PSF which can create phase shifts in the spectra of *J*-coupled metabolites that need to be corrected. While this can complicate quantitation, typically, singlets are most reliably fit in one-dimensional (1D) spectra, and they can only have real values of *a* [17]. As a result, the major metabolites that are typically quantified *in vivo* are not subject to complex PSFs and thus do not acquire additional phase shifts in the spectra.

## 3. Methods

Phantom scans were performed on a gray matter phantom containing physiological concentrations of metabolites. A $T_{1}$-weighted scan for localization was followed by three spectroscopic sequences: a regularly sampled EPSI sequence (${\mathrm{EPSI}}_{r}$), a doubly sampled EPSI sequence (${\mathrm{EPSI}}_{d}$), and a MEESPI sequence with 256 spectral points per TE (${\mathrm{MEEPSI}}_{256}$) resulting in a ${\mathrm{TE}}_{2}$ = 472.5 ms for a difference in echo time between the first and second readout of $\Delta {\mathrm{TE}}_{21}$ = 442.5 ms. The phase encoded resolution in the doubly sampled EPSI sequence was twice that of the regularly sampled EPSI sequence, and 512 spectral points per TR were sampled for each sequence. The following acquisition parameters were used by all three spectroscopic sequences: TE/TR=30/1500 ms, Analog to Digital Converter (ADC) bandwidth of 50 kHz resulting in a spectral bandwidth of 1190 Hz, a field of view (FOV) of 16 × 16 cm${}^{2}$, a slice thickness of 2 cm, and 8 averages per scan. The parameters that were changed between sequences are listed in Table 1. Water suppression was performed using the three-pulse WET sequence [18], applied just prior to the localization. As the use of echo-planar gradients is known to produce substantial eddy currents, a non-water-suppressed water scan was also collected to correct for the eddy current distortions [19]. Baseline distortions due to residual water signal were minimized using the WAVEWAT technique [20].

**Table 1:** Varying phantom scan parameters. Fixed parameters were: TE/TR=30/1500 ms, FOV = 16 × 16 cm${}^{2}$, slice thickness = 2 cm, 8 averages, and ${\mathrm{TE}}_{2}$ = 472.5 ms in ${\mathrm{MEEPSI}}_{256}$.

Scan	Grid	Voxel Size	Readout Points	Scan Time
${\mathrm{EPSI}}_{r}$	16×8	4 cm${}^{3}$	512	1 m 42 s
${\mathrm{EPSI}}_{d}$	16×16	2 cm${}^{3}$	512	3 m 18 s
${\mathrm{MEEPSI}}_{256}$	16×16	2 cm${}^{3}$	256	1 m 42 s

For the *in vivo* brain scans, a $T_{1}$-weighted image was used for localization prior to the following four spectroscopic sequences: a doubly sampled EPSI sequence (${\mathrm{EPSI}}_{d}$) with 512 spectral points per TR, and three separate MEEPSI sequences (${\mathrm{MEEPSI}}_{256}$, ${\mathrm{MEEPSI}}_{128}$, and ${\mathrm{MEEPSI}}_{64}$) with different values of $\Delta {\mathrm{TE}}_{21}$ and therefore different acquired spectral points but the same scan parameters otherwise. All scans used a TE/TR=30/1500 ms, an ADC bandwidth of 50 kHz resulting in a spectral bandwidth of 1190 Hz, a field of view (FOV) of 24×24 cm${}^{2}$, a grid size of 24×24, a slice thickness of 2.5 cm, which resulted in a voxel volume of 2.5 cm${}^{3}$, and 8 averages per scan. The scan parameters that were changed between sequences are listed in Table 2. As with the phantom scans, a non-water-suppressed scan was performed prior to the water-suppressed scan for use in eddy current correction.

**Table 2:** Varying *in vivo* scan parameters. Fixed parameters were: ${\mathrm{TE}}_{1}/\mathrm{TR}=30/1500$ ms, grid = 24×24, FOV = 24×24 cm${}^{2}$, slice thickness = 2.5 cm, voxel size = 2.5 cm${}^{3}$, and 8 averages.

Scan	Readout Points	$\Delta {\mathrm{TE}}_{21}$	Scan Time
${\mathrm{EPSI}}_{d}$	512	N/A	4 m 54 s
${\mathrm{MEEPSI}}_{256}$	256	442.5 ms	2 m 30 s
${\mathrm{MEEPSI}}_{128}$	128	227.5 ms	2 m 30 s
${\mathrm{MEEPSI}}_{64}$	64	120 ms	2 m 30 s

All scans were performed on a Siemens 3T Trio-TIM scanner running the VB17a platform. Data processing according to Figure 2 was performed offline using home-built MATLAB scripts. Fitting was done using LCModel software with a home-developed basis set consisting of 21 GAMMA [21] simulated brain metabolite spectra including, in part, N-acetylaspartate (NAA), N-acetylaspartyl glutamate (NAAG), glutamate (Glu), glutamine (Gln), glutathione (GSH), myo-inositol (mI), free choline (Cho), glycerophosphorylcholine (GPC), phosphorylcholine (PCh), and creatine (Cr) along with eight broad lipid and macromolecule resonance peaks.

## 4. Results

### 4.1. Phantom Scans

**Figure 5:**
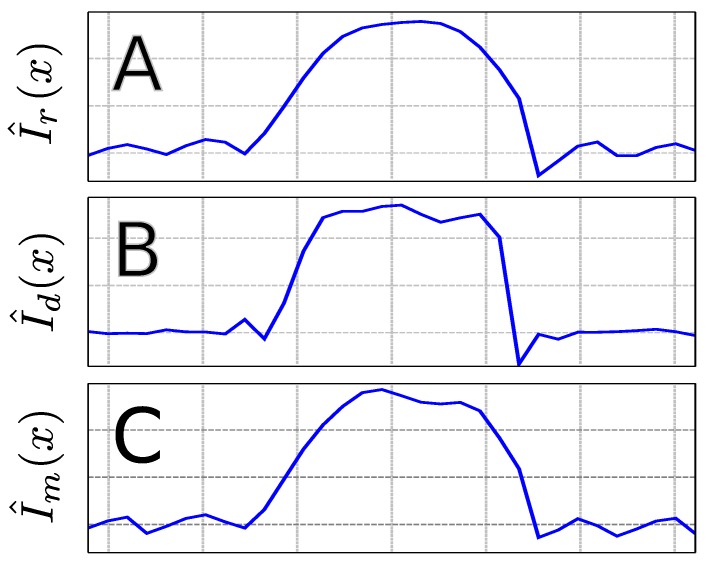
1D profiles along the phase encoded dimension for a select point along the readout dimension for the NAA singlet at 2.0 ppm for the (**A**) regularly sampled; (**B**) doubly sampled; and (**C**) multi-echo sampled *k*-space regions.

The collected phantom data was processed according to Figure 2 with no filters applied along the spatial dimensions in order to show the features of each PSF. All data sets were zero-filled to 32 points along the phase encoded dimension yielding an image data matrix size of 16 × 32. To analyze the effect of the multi-echo on the spatial profile, 1D profile maps were constructed by integrating the NAA singlet at 2.0 ppm. The 1D profiles, $\hat{I}\left(x\right)=I\left(x\right)*h\left(x\right)$, are simply a convolution of the true rectangular profile, I(x), with the respective PSF, h(x). The 1D profiles along the phase encoded dimension for a selected readout point are shown in Figure 5. Figure 5(A) shows the regularly sampled NAA profile, ${\hat{I}}_{r}\left(x\right)$, and because of the low resolution, the overall rectangular profile appears rounded. The effects of Gibbs ringing can be seen since no spatial filters were applied prior to application of the FFT along the spatial dimensions [22]. A higher resolution from doubling the *k*-space coverage can be seen in ${\hat{I}}_{d}\left(x\right)$ in Figure 5(B). While there is still significant Gibbs ringing, it can be seen that the rectangular shape of I(x) is much more pronounced due to the improved resolution. In Figure 5(C), it is shown ${\hat{I}}_{m}\left(x\right)$ that possess features between those of ${\hat{I}}_{r}\left(x\right)$ and ${\hat{I}}_{d}\left(x\right)$.

### 4.2. *In Vivo* Scans

**Figure 6:**
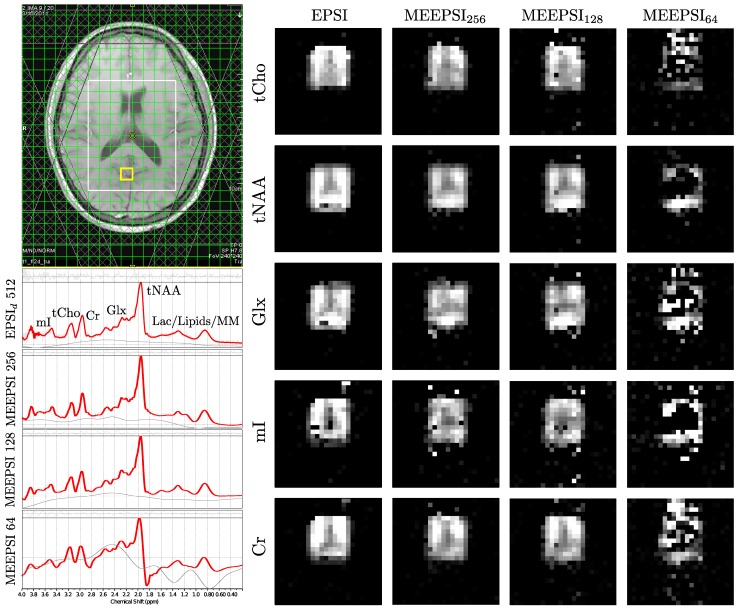
Comparison of fitted LCModel spectra and metabolite maps of a doubly sampled EPSI sequence with different MEEPSI readout lengths. The 2.5 mL spectra are taken from the location marked by the yellow box in the $T_{1}$-localized image. The metabolite maps were constructed from the LCModel fitted values with CRLBs <30%.

Results from an *in vivo* brain scan for the EPSI and MEEPSI sequences are shown in Figure 6. The green box shows the 24×24 imaging grid, and the white box shows the PRESS-excited VOI. Spectra were taken from a select 2.5 mL voxel in the gray matter of the occipital lobe, marked by the yellow box in the $T_{1}$-weighted localizing image. Metabolite maps were constructed for both EPSI and the different MEEPSI scans by taking the fitted concentrations from LCModel with Cramér Rao Lower Bounds (CRLBs) <30%. The LCModel-fitted spectrum for the EPSI sequence was compared with the three MEEPSI spectra acquired with different $\Delta {\mathrm{TE}}_{21}$ times and therefore different numbers of spectral readout points. The spectral dimension of each scan was zero-filled to 1024 points prior to LCModel fitting. It can be seen that despite the reduced spectral resolution due to the reduced number of collected points, the two MEEPSI scans with 256 and 128 spectral points had very similar LCModel fits to that of EPSI with 512 spectral points. As the number of points was further reduced to 64, it can be seen that LCModel struggled with baseline distortions and phase errors and consequently failed to produce a reliable metabolite map.

The central 6 × 8 voxels of the excited volume for each sequence shown in Figure 6 were fit with LCModel. Concentrations were expressed as ratios with respect to Cr and averaged across the volume. Mean ratios and coefficients of variation (CVs) among the voxels were computed and are shown in Table 3 for select metabolites. Only those metabolites fit with CRLBs <20% [23] were included in the calculations.

**Table 3:** Summary of the LCModel fit results for select metabolites for the different sequences. The central 6 × 8 voxel excited volume were processed. Ratios are shown with respect to Cr. $N_{inc}$ is the number of voxels in which LCModel reported a CRLB<20%. Only those voxels were included in the computation of mean ratios and coefficients of variation (CV). tCho = Cho + GPC + PCh, tNAA = NAA + NAAG, Glx = Glu + Gln.

	${\mathrm{EPSI}}_{d}$			${\mathrm{MEEPSI}}_{256}$			${\mathrm{MEEPSI}}_{128}$			${\mathrm{MEEPSI}}_{64}$		
	Mean Ratio	CV(%)	$N_{inc}$	Mean Ratio	CV(%)	$N_{inc}$	Mean Ratio	CV(%)	$N_{inc}$	Mean Ratio	CV(%)	$N_{inc}$
NAA	2.093	31.7	48	1.879	28.3	48	1.987	26.3	48	2.146	39.3	22
Glu	1.339	38.1	38	1.295	34.2	48	1.448	34.4	46	2.623	48.7	17
GSH	0.316	21.1	8	0.296	28.4	43	0.298	25.8	23	1.269	55.8	9
mI	0.631	31.4	37	0.726	27.2	47	0.681	26.7	47	2.401	90.6	20
tCho	0.338	17.5	47	0.310	20.8	48	0.341	43.4	47	0.334	76.2	25
tNAA	2.139	33.0	48	1.918	27.5	48	2.003	27.2	48	2.420	49.4	27
Glx	1.964	33.8	45	1.740	35.1	48	1.801	40.8	46	2.605	45.1	22

As can be seen in Table 3, the mean concentration ratios for each metabolite estimated by LCModel for ${\mathrm{EPSI}}_{d}$, ${\mathrm{MEEPSI}}_{256}$, and ${\mathrm{MEEPSI}}_{128}$ are all within 10% of each other, which indicates the estimate precision across the VOI is not greatly affected by mixing spin echoes with multiple TEs that have echo trains as short as 128 spectral points. However, the mean concentration ratios for ${\mathrm{MEEPSI}}_{64}$ deviate from the other scan estimates by as much as a factor of four (e.g., GSH, mI), which indicates the lower spectral resolution and possible FID truncation by using only 64 spectral points degrades the estimate precision greatly. Despite anatomical variations in the brain that may affect metabolite concentrations, such as voxels primarily consisting of cerebral spinal fluid (CSF), the CV of each metabolite estimate for ${\mathrm{MEEPSI}}_{128}$ is less than 50%, and the CVs for ${\mathrm{EPSI}}_{d}$ and ${\mathrm{MEEPSI}}_{256}$ are all less than 40%. If a method existed to unbiasedly exclude voxels primarily consisting of CSF and taking partial volume effects into account, the CVs would be expected to decrease. However, the CVs of estimate ratios for ${\mathrm{MEEPSI}}_{64}$ were much higher for each metabolite, with only NAA having a CV less than 40% and mI and tCho having CVs greater than 75%. Again, this indicates a decrease in the precision and reproducibility of the metabolite ratios estimated by LCModel for echo trains shorter than 128 points. The number of voxels successfully fit with a CRLBs<20% in ${\mathrm{MEEPSI}}_{64}$ is less than half as many as those in the other three sequences. ${\mathrm{MEEPSI}}_{256}$ and ${\mathrm{MEEPSI}}_{128}$ fit a comparable number of voxels per metabolite to ${\mathrm{EPSI}}_{d}$, with the exception of GSH, where ${\mathrm{EPSI}}_{d}$ is actually inferior to the MEEPSI scans.

## 5. Discussion

As seen in Figure 5(A), the 1D NAA spatial profile for the regularly sampled phase encoded dimension shows a rounded profile due to the low resolution (8 samples zero-filled to 32). Gibbs ringing, originating from the lobes of $h_{r}\left(x\right)$, can be seen to extend beyond the VOI. There is also an apparent asymmetry in the profile that can arise from a variety of possible factors that are difficult to control experimentally. Shimming is performed over the entire VOI, so while the overall shimmed line-width may be acceptable, it is not expected to be uniform over the entire volume, leading to spatially dependent line-widths. Such variations in the magnetic field can result in differences in the local water-suppression and other baseline distortions that can complicate the simple method of peak integration used to create the 1D profiles. Regardless, the same asymmetry is present when the resolution is doubled in ${\hat{I}}_{d}\left(x\right)$ as seen in Figure 5(B) (16 samples zero-filled to 32). With twice the resolution, the true rectangular profile from the PRESS localization is much more realized than with the lower resolution profile in ${\hat{I}}_{r}\left(x\right)$. The Gibbs ringing present in ${\hat{I}}_{d}\left(x\right)$ appears to oscillate more rapidly than in ${\hat{I}}_{r}\left(x\right)$, which is expected when comparing the oscillation frequencies of $h_{r}\left(x\right)$ and $h_{d}\left(x\right)$ (Equation (5) and Equation (7)).

Equation (17) and Figure 4 indicate that the 1D profile along the phase encoded dimension, ${\hat{I}}_{m}\left(x\right)$, is expected to resemble both ${\hat{I}}_{r}\left(x\right)$ and ${\hat{I}}_{d}\left(x\right)$ as $h_{m}\left(x\right)$ is a weighted sum of $h_{r}\left(x\right)$ and $h_{d}\left(x\right)$. As can be seen in Figure 5(C), ${\hat{I}}_{m}\left(x\right)$ appears to resemble both ${\hat{I}}_{r}\left(x\right)$ and ${\hat{I}}_{d}\left(x\right)$ in shape and structure. The lower resolution of ${\hat{I}}_{r}\left(x\right)$ that produced the overall rounded shape can be seen, but at the same time, the presence of more rectangular features that are more characteristic of ${\hat{I}}_{d}\left(x\right)$ can be seen as well. This mixture of the two different features, as predicted by Equation (17) implies an improved spatial resolution over ${\hat{I}}_{r}\left(x\right)$ as demonstrated by the narrowing of the FWHM of the PSF as shown in Figure 4(D).

The $T_{2}^{*}$ times in the gray-matter phantom were rather long, leading to a sharp NAA singlet that could be integrated easily. The long $T_{2}^{*}$ times required longer readouts in order to prevent clipping of the signal that would either lead to spectral ringing or require line-broadening filters. The longer required readouts lengthen $\Delta {\mathrm{TE}}_{21}$, limiting the extent of the improved spatial resolution according to Equation (9). The situation changes with *in vivo* scans, where the tissue heterogeneity leads to drastically shorter $T_{2}^{*}$ times. Despite the well-known Fourier relationship that sampling more time points improves spectral resolution, it has been shown that sampling points after the signal has sufficiently decayed does not improve the spectral resolution [24]. As a result, the *in vivo* readout duration can be significantly reduced to improve the spatial resolution at no expense to spectral resolution.

As can be seen in Figure 6, reducing the length of the readout has little effect on the ability to fit the major metabolites using LCModel. Even acquiring as few as 128 spectral points, the fitted spectrum does not suffer from any visible loss of spectral resolution, and the metabolite maps produced from the LCModel concentration estimates show similar spatial distributions to EPSI. As the number of spectral points is reduced to 64, it is shown that LCModel struggles to consistently produce well fit spectra, resulting in unreliable and often inaccurate metabolite maps. While the peaks for the same major metabolites are still visible (NAA, Cr, Cho, Glx, mI), there appears to be a severe baseline distortion as well as other frequency-specific phase errors. As LCModel does not apply any filters, the distortions could also be the result of the FID not being fully decayed at the end of the acquisition, resulting in spectral ringing after the FFT. The reduction of $\Delta {\mathrm{TE}}_{21}$ (and subsequent improvement in spatial resolution) is thus limited by the fact that the signal should have sufficiently decayed before applying the $180^{\circ }$ pulse and collecting the next spin-echo.

The results in Table 3 indicate that improved fitting is achieved with the ${\mathrm{MEEPSI}}_{256}$ sequence compared to the doubly sampled ${\mathrm{EPSI}}_{d}$ when looking at the notably higher number of fitted voxels for GSH. However, this interpretation is somewhat misleading, as the source of this increase is due to the reduced noise in the ${\mathrm{MEEPSI}}_{256}$ sequence and the chosen CRLB cutoff. Due to the broadened PSF of the ${\mathrm{MEEPSI}}_{256}$ sequence, its real voxel size is larger than that of ${\mathrm{EPSI}}_{d}$ and experiences more partial voluming. Because of the homogeneity of the healthy brain, the larger voxels do indeed have higher SNR, resulting in lower CRLBs. In the case of ${\mathrm{EPSI}}_{d}$, many of the voxels had CRLBs for GSH just slightly above the threshold of 20%, indicating that the slight increase in SNR from the larger voxel size in ${\mathrm{MEEPSI}}_{256}$ helped fit more voxels with CRLBs lower than the threshold. A glance at the CVs in the table shows little actual difference in the fit reliability between the two sequences.

The extent by which the resolution is improved in MEEPSI relative to the regularly sampled ${\mathrm{EPSI}}_{r}$ can be difficult to determine experimentally. Using the definition of *a* in Equation (9), and the determined PSF in Equation (17), the real voxel size can be estimated for each metabolite based on the overall improvement of spatial resolution. Given the reported $T_{2}$ values for NAA, Cr and Cho [25], the values for *a* and improvement in spatial resolution are estimated and summarized in Table 4. As expected, the least amount of improvement comes with ${\mathrm{MEEPSI}}_{256}$ (where $\Delta {\mathrm{TE}}_{21}\gg T_{2}$ for most metabolites), with improvement in spatial resolution ranging from 10% to 50% depending on the metabolite. For ${\mathrm{MEEPSI}}_{128}$ and ${\mathrm{MEEPSI}}_{64}$, it can be seen that $\Delta {\mathrm{TE}}_{21}∼T_{2}$, and so dramatic improvements in spatial resolution can be seen. Of course, the estimated values in Table 4 are only approximate as the values of $T_{2}$ are known to vary by pathology as well as other factors. For *J*-coupled resonances, the analysis becomes more complicated as the value for *a* can take on complex values. However, since most of the major metabolites are fitted based on singlet resonances, the overall performance of the fitting is not expected to deteriorate dramatically. Nevertheless, the major *J*-coupled metabolites still managed to be fit albeit with higher CVs, as is to be expected with the more complicated PSF.

**Table 4:** Summary of the estimated values of *a* and the corresponding relative improvement in spatial resolution (ΔFWHM) for the singlets of the major metabolites: NAA, Cr, and Cho.

	$\Delta {\mathrm{TE}}_{21}$	NAA, $T_{2}\approx 275$ ms		Cr (3.03), $T_{2}\approx 150$ ms		Cr (3.92), $T_{2}\approx 130$ ms		Cho, $T_{2}\approx 195$ ms	
		a	ΔFWHM	a	ΔFWHM	a	ΔFWHM	a	ΔFWHM
${\mathrm{MEEPSI}}_{256}$	442.5 ms	0.20	∼50%	0.05	∼15%	0.03	∼10%	0.10	∼29%
${\mathrm{MEEPSI}}_{128}$	227.5 ms	0.44	∼77%	0.22	∼53%	0.17	∼44%	0.31	∼65%
${\mathrm{MEEPSI}}_{64}$	120.0 ms	0.65	∼89%	0.45	∼78%	0.39	∼73%	0.54	∼84%

## 6. Conclusions

We have shown theoretically and experimentally that the use of multiple phase encoded spin echoes within a single TR can improve upon the spatial resolution in an EPSI experiment, without any added experimental time. While this study focused on only two echoes, there is nothing preventing the application of additional echoes. In the case of more echoes, the same theoretical approach in determining the PSF could be used to estimate the degree of improved spatial resolution. The fact that $T_{2}^{*}$ times *in vivo* are relatively short allows for shorter readouts to be acquired at no expense of spectral resolution, reducing the overall effect of irreversible $T_{2}$ decay on the spatial profiles. This MEEPSI sequence has been demonstrated on the brain of a healthy volunteer in a clinical setting and is shown to be useful in improving the spatial resolution (which can be used to reduce overall scan time) of other spectroscopic imaging sequences as well.
